# Development and Characterization of Magnetic Nanoemulsion-Based Senolytic Peptides for Osteoarthritis Treatment

**DOI:** 10.3390/ijms26031292

**Published:** 2025-02-03

**Authors:** Camelia-Mihaela Zara-Danceanu, Jenifer García-Fernández, Dumitru-Daniel Herea, Daniel Gherca, Irene de Francisco Carrera, Luminita Labusca, Maria de la Fuente

**Affiliations:** 1 National Institute of Research and Development in Technical Physics, 700050 Iasi, Romania; cdanceanu@phys-iasi.ro (C.-M.Z.-D.); dherea@phys-iasi.ro (D.-D.H.); dgherca@phys-iasi.ro (D.G.); 2 Transcend Ceter Regional Oncology Institute, 700483 Iasi, Romania; 3 Nano-Oncology and Translational Therapeutics Group, Health Research Institute of Santiago de Compostela (IDIS), Servizo Galego de Saúde (SERGAS), 15706 Santiago de Compostela, Spain; Jenifer.Garcia.Fernandez@sergas.es (J.G.-F.); irene.de.francisco.2@gmail.com (I.d.F.C.); 4 Orthopedics and Trauma Clinic, County Emergency Hospital, 700111 Iasi, Romania; 5 Biomedical Research Networking Center on Oncology (CIBERONC), Instituto de Salud Carlos III, 28029 Madrid, Spain; 6 DIVERSA Technologies S.L., Edificio Emprendia, 15782 Santiago de Compostela, Spain

**Keywords:** osteoarthritis, senolytic peptides, nanoemulsions, drug delivery, magnetic nanoparticles

## Abstract

The formulation and characterization of a novel nanoemulsion (NE) delivery system for senomodulator peptides aimed at enhancing the treatment of osteoarthritis (OA) are reported, in combination with magnetic nanoparticles (MNPs), for improving targeted delivery and traceability. Osteoarthritis, a prevalent degenerative joint disease associated with aging, is currently not effectively treated by disease-modifying therapies, posing a consistent health burden on individuals and healthcare systems worldwide. Existing treatments, such as nonsteroidal anti-inflammatory drugs and intra-articular injections, are limited by inadequate local drug concentrations and rapid clearance, often necessitating costly joint replacement. Lipid-based NE composed of biocompatible and biodegradable vitamin E and sphingomyelin, associated with the senolytic peptide NE:TUB1, is able to target senescent cells implicated in OA progression. Improved cellular retention and therapeutic effects of the associated TUB1 peptide, compared to its free form, have been demonstrated, suggesting a significant enhancement in therapeutic potential. The incorporation of MNPs to obtain NE:TUB1-MNP formulations offers the advantage of being traceable in vivo through clinically available imaging technologies, with the potential to enhance targeting capabilities through magnetic guidance. The characterization of NE:TUB1-MNPs involved the assessment of their physical and chemical properties, interaction with cells, cytotoxicity profile, and nanoparticle uptake in vitro using human primary adipose-derived stem cells. NE and NE:TUB1-MNP are shown to be stable, non-toxic, and capable of efficient intracellular uptake. The inclusion of MNPs not only supports cell viability and proliferation but also facilitates medium and long-term product traceability within joints, offering a promising approach for localized treatment. The enhanced anti-senescent role of NE:TUB1-MNP formulations are highlighted, suggesting their potential utility in mitigating OA progression and possibly other degenerative diseases. In conclusion, the study presents a novel therapeutic approach for OA, NE:TUB1-MNPs, leveraging the synergistic effects of peptide-functionalized nanoemulsions and magnetic nanoparticles to improve targeted delivery and therapeutic outcomes. This innovative formulation could pave the way for new treatments for OA and other joint-related conditions, offering significant advancements in regenerative medicine.

## 1. Introduction

Osteoarthritis (OA) is a pervasive musculoskeletal disorder characterized by the degeneration of joint cartilage and underlying bone, resulting in pain, stiffness, and impaired physical function [1]. As one of the most prevalent joint diseases globally, OA exerts a substantial burden on individuals, healthcare systems, and economies [2]. This chronic condition, often associated with aging, presents a multifaceted challenge impacting public health, disability, and quality of life [3].

From an epidemiology perspective, around 240 million people worldwide suffer from symptomatic OA, with prevalence increasing sharply with age. It is estimated that approximately 10% of men and 18% of women over the age of 60 suffer from symptomatic OA, particularly in the knees and hips [4]. The condition is more common in women, especially post-menopausal women, and its incidence is expected to rise due to increasing life expectancy and rising obesity rates—a major modifiable risk factor for OA [5]. Furthermore, OA is responsible for a significant proportion of global disability-adjusted life years (DALYs), contributing to a growing public health burden, particularly in developed and rapidly aging societies [6].

The burden of OA is expected to increase, necessitating a comprehensive understanding of its epidemiology, risk factors, and socio-economic implications [7]. Currently, there is no disease-modifying therapy for OA, and early stages are managed with symptom-based approaches aimed at alleviating pain and improving function. Pharmacologic strategies, such as the administration of nonsteroidal anti-inflammatory drugs, can provide pain relief, but their efficacy is limited by low local concentrations within the joints. Intra-articular (IA) injections of corticosteroids and hyaluronate offer alternative therapeutic options for managing OA pain and symptoms. However, the effectiveness of these injections is debated, partly due to the rapid clearance of the drugs from the joints and the dense extracellular matrix in cartilage that hinders drug absorption [8].

As OA progresses, many patients find conventional treatments insufficient, often necessitating total joint replacement [9]. While effective in restoring function and alleviating pain, joint replacement is a costly intervention requiring specialized surgical expertise, posing a significant economic challenge [10]. The lack of a pharmaceutical or therapeutic breakthrough capable of halting or reversing the degenerative process, especially in younger patients, underscores the need for further research and innovation in OA treatment.

OA is currently perceived as a whole joint disease that progressively affects not only cartilage but also the subchondral bone, synovial layer, periarticular muscles and tendons, as well as ligaments and menisci depending on joint anatomy. Articular cartilage from OA patients accumulates dedifferentiated and senescent cells, along with increased production of pro-inflammatory cytokines and catabolic enzymes, leading to the breakdown of cartilage extracellular matrix (ECM) and joint degeneration. Senescent cells resist apoptosis and release a proinflammatory secretome known as the senescence-associated secretory phenotype (SASP), which alters the structure and function of surrounding cells and tissues. Accumulation of senescent cells is implicated in the progression of various age-related diseases, including OA. In this context, the key role of transmembrane protein connexin43 (Cx43) for overactivity in senescence and cartilage degradation has already been demonstrated [11]. Clearing senescent cells or targeting SASP pathways has been reported to alleviate the symptoms of some age-related diseases. However, there remains a clear medical need for effective senotherapeutic agents that selectively induce the death of senescent cells and restore normal function. Pharmacological inhibitors targeting Cx43 protein interactions, such as therapeutic peptides, are emerging as promising drugs for various disorders, including cancer and neurodegeneration [12,13,14]. These peptides typically have specific amino acid sequences that enable them to selectively bind to and affect senescent cells, either by inducing apoptosis or by altering cellular pathways to reduce senescence-associated secretory phenotype (SASP) factors [15]. Physicochemical characteristics of senomodulator peptides include stability in biological environments, specific receptor affinity, and favorable pharmacokinetic properties that enhance their therapeutic efficacy. Beyond OA, senomodulator peptides have applications in other fields, such as oncology, where they are used to target senescent cancer cells, and dermatology, where they are explored for their anti-aging properties [16].

A specific peptide, TUB1, derived from Cx43, has shown promise in reducing the accumulation of senescent chondrocytes and triggering the re-differentiation of osteoarthritic chondrocytes, making it a potential senotherapeutic agent for OA and other aging-associated diseases [17]. The exploration of senomodulator peptides as a therapeutic avenue for treating degenerative diseases, including OA, holds significant promise [18,19,20]. A major challenge is ensuring that senotherapeutic agents reach target tissues in sufficient concentrations; improving bioavailability and developing targeted delivery methods are critical [12,21].

Recent remarkable developments in Drug Delivery Systems (DDSs) are rapidly impacting the field of IA administration [22]. In this sense, nanoemulsions offer efficient modalities for targeted drug delivery [23]. Our group has developed lipid-based nanoemulsions (NE) composed of biodegradable and biocompatible vitamin E, sphingomyelin, and a lipid-PEG to enhance surface hydrophilicity and facilitate functionalization. These nanoemulsions are safe-by-design formulations that present several advantages concerning other nanosystems, such as their simple composition, easy manufacturing conditions, long-term stability, and the versatility to incorporate additional targeting moieties and therapeutic payloads under demand, including peptides and nucleic acids [24,25,26,27]. The senolytic peptide TUB1 has been successfully associated with these NE, demonstrating improved retention time and therapeutic effects compared to the free peptide. In a reported study, the higher retention time on the target site of the peptide-encapsulated NE versus the free peptide was highlighted, leading to a significant improvement in the intrinsic therapeutic effects of the peptide. Thereby, the novel nanoemulsion formulation has already proved to be a successful DDS for IA injection [28].

Magnetic nanoparticles (MNPs) are versatile tools for improving targeted delivery and product traceability after in vivo delivery [29], with the advantages of increased sensitivity, better detection capabilities, and ease of operation have been studied for possible application of OA diagnosis [30]. Magnetic nanoparticles of various compositions and coatings have been recently tested for their versatility as Drug Delivery Systems for pharmacologic applications as well as for their use in regenerative medicine, including bone and cartilage [31]. Due to their composition, which is highly compatible with cell molecular mechanisms for iron handling and turnover, MNPs are proven non-toxic to cells and tissues in vitro and in vivo [32,33,34,35]. MNPs offer the advantage of being traceable in vivo using clinically available detection equipment (magnetic resonance imaging—MRI). Incoming potential clinically available detection methods such as magnetic particle imaging (MPI) or dual MRI-PET systems can further improve the accuracy, specificity as well as resolution of in vivo tracking systems [36]. Injectable therapeutics for local, regional or systemic applications consistently benefit the use of tracking methods, improving safety in administration as well as detection of time-dependent efficiency outcomes [37]. We previously developed a simple, biocompatible, and highly sensitive T2 MRI formulation by synthesizing manganese ferrite nanoparticles coated with vitamin E and encapsulating them in biocompatible nanoemulsions, demonstrating excellent in vivo biocompatibility and strong MRI signals both in vivo and ex vivo, highlighting their potential to enhance negative MR contrast and significantly improve MRI sensitivity for biomedical imaging [38]. In this study, we report the formulation and characterization of a novel senotherapeutic nanoformulation based on peptide-loaded NE (NE:TUB1) aimed at IA delivery for the treatment of OA. Two different strategies were proposed for an effective association of the TUB1 peptide: the establishment of electrostatic interactions and “click” reactions. For this, the TUB1 peptide was modified in the N-terminus with arginine residues (TUB1-Arg) and an azide group (TUB1-N_3_), as previously reported for surface binding of aptamers [39]) and proteins [40].

Furthermore, formulations of NE:TUB1-MNPs are prepared to enhance targeting and delivery capabilities through magnetic guidance. An in-depth analysis of NE:TUB1 and NE:TUB1-MNP is performed, aiming to describe their physical and chemical properties, interaction with cells, potential cytotoxicity, and nanoparticle uptake in vitro using human primary adipose-derived stem cells. This research aims to pave the way for in vivo validation and potentially offer a localized, effective treatment for OA.

## 2. Results

### 2.1. Physical and Chemical Characteristics of Peptide-Functionalized Nanoemulsions

Assessment of the particle size has been performed using a dynamic light scattering technique, followed by physical characterization in terms of stability (polydispersity index) as well as the assessment of surface charge. Results are shown in Table 1. It is remarkable how the surface charge changes from negative values for the blank NE and gets more positive when the peptides are associated, confirming the presence of the peptides (cationic/positively charged at neutral pH) on the surface of the nanosystem.

**Figure 1:**
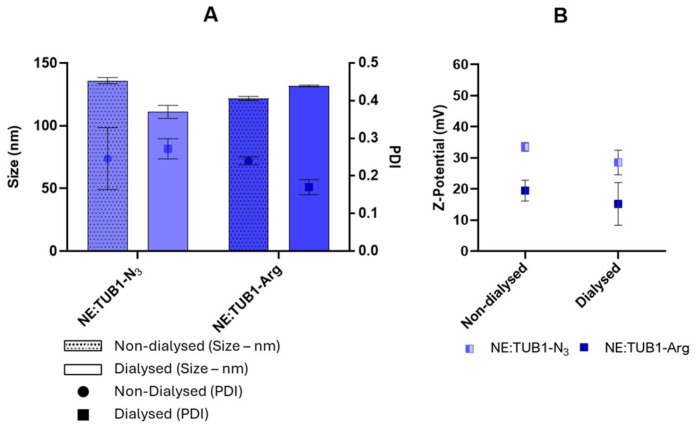
Physicochemical characterization of dialyzed and non-dialyzed NE:TUB1-Arg (dark blue) and NE:TUB1-N_3_ (light blue) in terms of size and polydispersity index (**A**) and surface charge or Z-Potential (**B**), measured by DLS.

**Figure 2:**
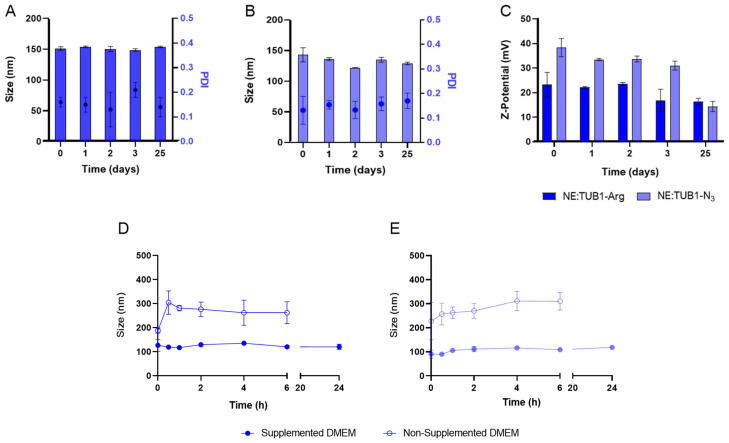
Colloidal stability determined in water (**A**–**C**) after storage at 4 °C for up to 25 days and during incubation in supplemented (10% serum) and non-supplemented cell culture media (DMEM) at 37 °C for up to 24 h (**D**,**E**). (**A**) Size (in bars) and polydispersity index (in dots) of peptide-functionalized NEs associated by electrostatic interactions (NE:TUB1-Arg). (**B**) Size (in bars) and polydispersity index (in dots) of peptide-functionalized NEs associated by “click” chemistry (NE-TUB1-N_3_). (**C**) Z-Potential stability of NE:TUB1-Arg and NE-TUB1-N_3_ for up to 25 days. (**D**) Size of NE-TUB1-Arg and (**E**) NE:TUB1-N_3_, incubated in supplemented (10% FBS) and non-supplemented DMEM.

As shown in Figure 1, these nanosystems were dialyzed and characterized once again by DLS (before and after dialysis) to assess if this mild process could impair the physicochemical properties of NEs. This method was selected to isolate the NE:TUB1 from the free peptide or other lipid components remaining in the media, as well as to determine the AE% of the peptide. DLS analysis showed that neither particle size or PDI (Figure 2A) nor surface charge (Figure 2B) were affected during the dialysis process.

Evaluating the stability of the resulting nanosystems over time and in different biological media is essential to predict their fate, especially after administration. Storage stability at 4 °C was studied, and the analysis by DLS showed that NE:TUB1-Arg (152 ± 2 nm, PDI = 0.16, ZP = +20 ± 2 mV) and NE:TUB1-N_3_ (133 ± 4 nm, PDI = 0.2, ZP = +30 ± 2 mV) were stable at least for 25 days (Figure 2A–C).

In addition, the stability of the peptide-loaded nanoemulsions was determined in biological media (non-supplemented and supplemented DMEM with 1% FBS). According to DLS measurements and optical visualization of the samples after incubation with these conditions, it was possible to observe that both NE:TUB1-Arg (Figure 2D) and NE:TUB1-N_3_ (Figure 2E) were stable in the cell culture medium containing fetal bovine serum (FBS). This could be related to the adsorption of serum proteins to the surface of the positively charged nanosystems through electrostatic interactions, which leads to improved dispersion stability as previously reported in other studies [34] but is not indicative of aggregation as they remain stable within time.

Regarding the AE% of the peptide TUB1 to NE, determined by HPLC after dialysis, the results showed an AE of 63.3 ± 5.9% for the “click” conjugation and a 60.2 ± 10.9% for the peptide associated with electrostatic interactions, indicating that both methods are comparably effective for incorporating the peptide to the NE, achieving a similar association capacity.

### 2.2. Characterization of the Magnetic Formulations

**Figure 3:**
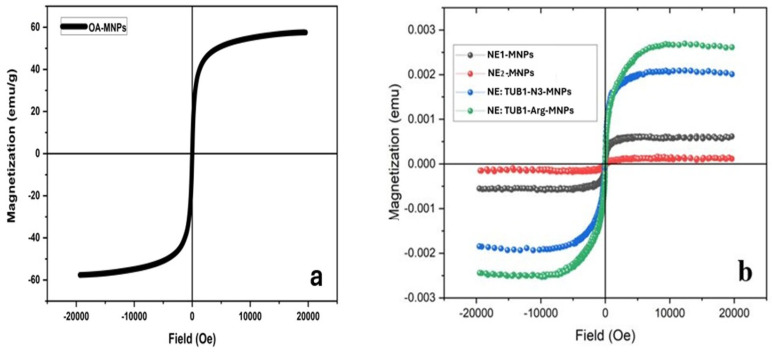
Hysteresis curves at room temperature for MNPs (**a**) and for NE−MNPs (VE:SM:PEG2k (black), VE:SM:PEG2k−DBCO (red), NE:TUB1−N_3_−MNPs (blue), and NE:TUB1−N_3_−MNPs (green) (**b**).

The magnetization curve of MNPs (Fe_3_O_4_ magnetic nanoparticles with oleic acid) is shown in Figure 3a, showing superparamagnetic behavior with a saturation magnetization of 58 emu/g. Measured magnetization was determined at room temperature and depended on the applied field (−20 to 20 kOe). Comparatively, hysteresis curves for magnetic formulations with and without peptides are shown in Figure 3b. It can be observed that NE:TUB1-MNPs maintain MNPs’ magnetic properties.

**Figure 4:**
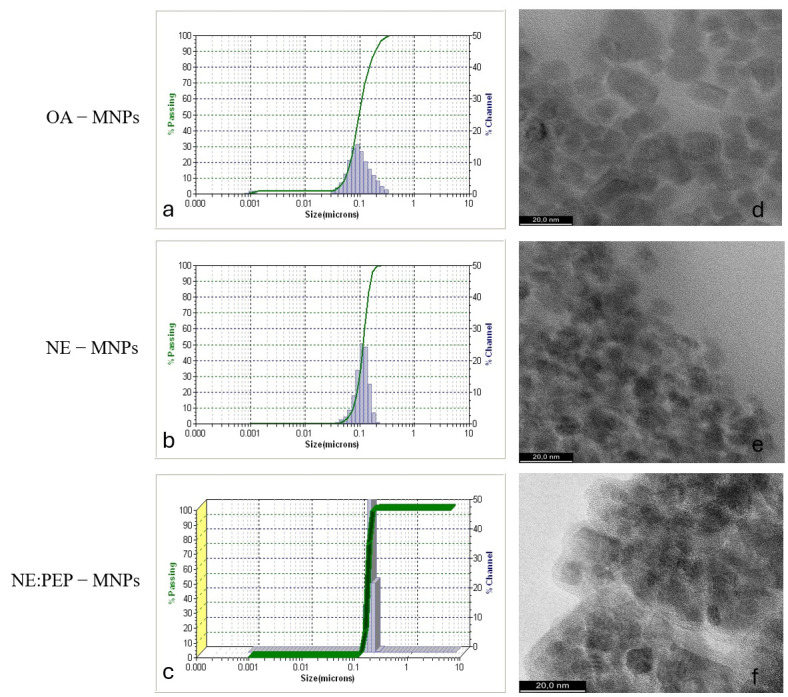
Dynamic light scattering and ultrahigh-resolution transmission electron microscopy (UHR-TEM) images of magnetic nanoparticles and magnetic NE: (**a**) DLS for as-prepared MNPs; (**b**) DLS for NE-MNPs; (**c**) DLS for NE:TUB1-MNPs; (**d**) TEM for MNPs; (**e**) TEM for NE-MNPs and (**f**) TEM for NE:TUB1-MNPs.

Magnetic nanoparticles were characterized for size by using a dynamic light scattering method (DLS-Microtrac/Nanotrac 252, Microtrac, Montgomeryville, PA, USA) and ultra-high-resolution transmission electron microscopy imaging (UHR-TEM LIBRA^®^200MC, Carl Zeiss GmbH, Aalen, Germany) (Figure 4).

### 2.3. FTIR Analysis of Magnetic Nanoformulations

The FT-IR spectra obtained for the nanoformulation containing NE:TUB1 exhibit several prominent peaks at specific wavenumbers (e.g., 3743 cm^−1^, 3423 cm^−1^, 3265 cm^−1^, 2955 cm^−1^, 2921 cm^−1^, 2851 cm^−1^, 1740 cm^−1^, 1636 cm^−1^, 1543 cm^−1^, 1456 cm^−1^, 1395 cm^−1^, 1024 cm^−1^, 671 cm^−1^, and 583 cm^−1^, respectively. These peaks provide valuable information regarding the molecular structure and functional groups present in the formulation.

**Figure 5:**
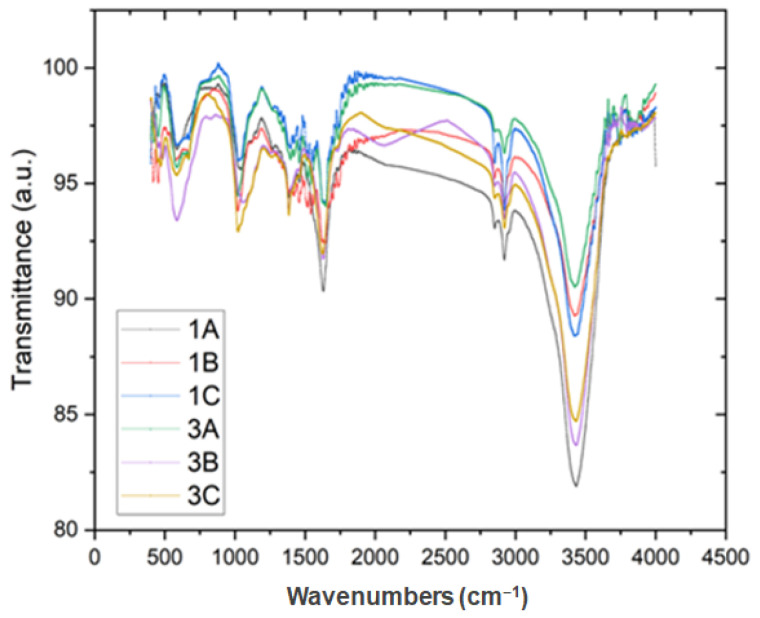
FT-IR spectra of NE:TUB1-N_3_ -MNPs for three different batches (1A: black line; 1B: red line; 1C: blue line) and NE:TUB1-Arg-MNPs for three different batches (3A: magenta line; 3B: cyan line; 3C: orange line).

The presence of a strong peak at 3743 cm^−1^ suggests the existence of hydrogen bonding, possibly involving hydroxyl (-OH) groups or -N-H stretching vibrations. This indicates the presence of functional groups that can interact with other molecules or within the nanostructure. The peak at 3423 cm^−1^ indicates the presence of another hydrogen bonding interaction, possibly involving amine (-NH) or amide (N-H) groups. This peak is commonly observed in proteins and peptides due to the stretching vibrations of these functional groups. The peak at 3265 cm^−1^ further supports the presence of N-H stretching vibrations, indicating the involvement of amide groups. The peaks at 2955 cm^−1^ and 2851 cm^−1^ correspond to C-H stretching vibrations, indicating the presence of aliphatic hydrocarbon chains. These peaks suggest the presence of organic compounds—lipids or surfactants—used in the formulation. The peak at 1740 cm^−1^ corresponds to the stretching vibrations of carbonyl (C=O) groups. This peak indicates the presence of functional groups such as esters, ketones, or carboxylic acids in the nanoformulation. The 1636 cm^−1^ peak corresponds to the presence of aromatic rings or conjugated double bonds. This peak can be attributed to the aromatic amino acids present in the peptide TUB1 or other aromatic compounds in the formulation. The peaks at 1543 cm^−1^, 1456 cm^−1^, and 1395 cm^−1^ can be assigned to the bending vibrations of N-H or C-N bonds in amide groups. The peak at 1024 cm^−1^ corresponds to the stretching vibrations of C-O bonds, indicating the presence of ether or ester groups. This finding suggests the presence of additional functional groups or excipients in the nanoformulation. The peaks at 671 cm^−1^ and 583 cm^−1^ can be attributed to the bending vibrations of metal–oxygen bonds (Figure 5). These peaks are particularly interesting, as they indicate the presence of Fe_3_O_4_ nanoparticles in the nanoformulation. All these peaks provide descriptive information about the composition and structural features of the nanoformulation, highlighting the presence of peptides, hydroxyl groups, amide groups, aliphatic hydrocarbons, carbonyl groups, aromatic compounds, and metal–oxygen interactions.

### 2.4. Cytotoxicity Assays

**Figure 6:**
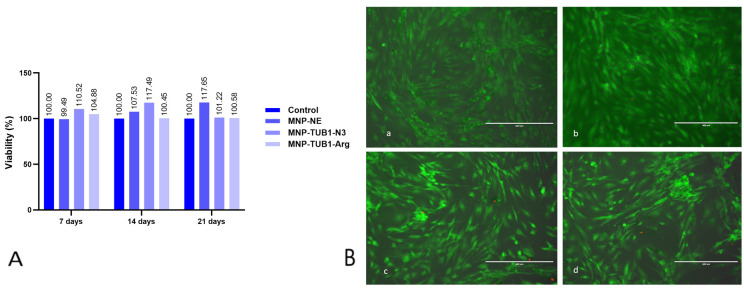
ADSC viability in the presence of various nanoformulations: NE-MNPs, NE:TUB1-N_3_-MNPs, and NE:TUB1-Arg-MNPs. (**A**) Viability assessment was performed using serial MTT assay at 7, 14, and 21 days after exposing cells with respective formulations. (**B**) LIVE/DEAD fluorescence at 7 days exposure. EVOS fluorescence inverted microscope 10× magnification, stacked images of bright field (BF), GFP, and RFP. (**a**) ADSC control; (**b**) ADSC incubated with MNPs; (**c**) ADSC incubated with NE:TUB1-N_3_-MNPs; (**d**) ADSC incubated with NE:TUB1-Arg-MNPs. Please note very few red elements in (**c**,**d**) demonstrating overall good viability (green cells).

The assessment of ADSC viability under exposure to nanoformulations was determined using an MTT assay. For all the tested formulations, the control formulation (without peptide encapsulation) NE-MNPs and the peptide-loaded formulations (NE:TUB1-N_3_-MNPs and NE:TUB1-Arg-MNPs) showed no cytotoxicity effect against ADSC with reported cell viabilities overcoming 95%, compared to untreated controls, after 48 h, 14 and 21 days of exposure. MTT assay is a colorimetric assay used to measure cellular metabolic activity as an indicator of cell viability, proliferation, and cytotoxicity. It is based on the ability of functional cellular mitochondrial dehydrogenase enzymes to be efficient in reducing a yellow tetrazolium salt (3-(4,5-dimethylthiazol-2-yl)-2,5-diphenyltetrazolium bromide) to a purple formazan product. The test represents an indirect indicator of cell viability as well as potential cell proliferation (Figure 6A).

Similar results were obtained using LIVE/DEAD assay. LIVE/DEAD is a fluorescence-based assay designed to distinguish live and dead cells based on differences in membrane integrity and intracellular components. Live cells exhibit a characteristic fluorescence when stained with a dye called calcein AM, which is cleaved by intracellular esterase to yield green fluorescence. Dead cells with damaged membranes take up ethidium homodimer-1, which intercalates with nuclear DNA and emits red fluorescence. Cells showed good viability compared to non-treated controls, with very few red elements appearing in the field of view (FOV) (Figure 6B).

### 2.5. Quantitative Assessment of MNPs Upload by ADSC-Ferrozine Assay

**Figure 7:**
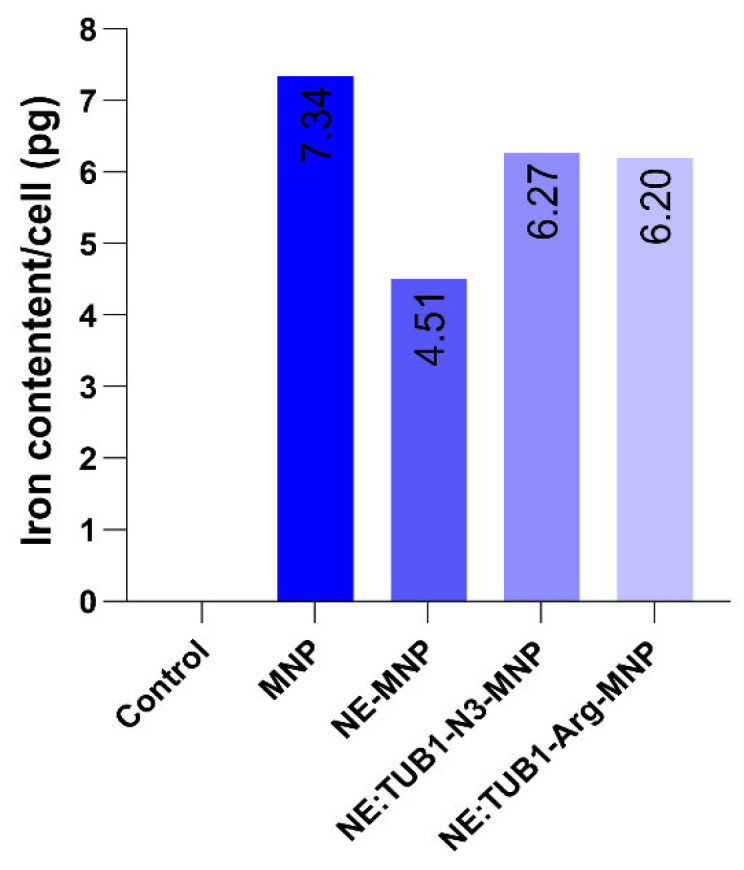
Iron content per cell (picograms) detected using ferrozine assay after 48 h interaction with cells.

The intracellular iron concentration normalized against the cell number of replicate wells is presented in Figure 7 The average amount of uploaded iron was 5.5 picograms/cell with a (maximum 6.27 for NE:TUB1-N_3_-MNP and minimum 4.51 for NE-MNP (without peptides). Nanoemulsion presence non-significantly decreases iron particle upload compared to MNP only. Higher amounts of NE-MNPs could be internalized when functionalized with peptides compared to NE only (Figure 7).

### 2.6. Morphological Aspect and Senescence Assay

**Figure 8:**
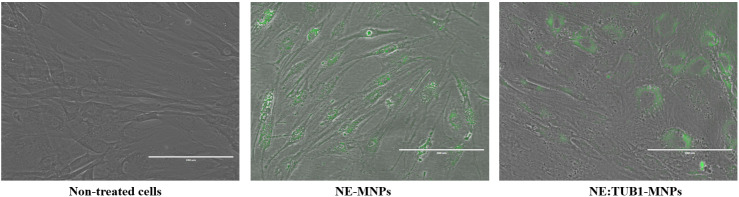
Fluorescence images of ADSC cells treated with control NE-MNPs and NE:TUB1-X-MNPs ADSC (control (untreated); ADSC incubated with NE-MNPs; ADSC incubated with NE:TUB1-MNPs.

Fluorescent-labeled nanoformulations could be observed under a fluorescent inverted microscope, demonstrating their intracellular localization. Cells were shown to retain morphologic features in terms of shape, distribution within the culture dish, and agglomeration, showing no apparent differences compared to unloaded controls. Cell membrane as well as nuclear membrane integrity could be demonstrated as well (Figure 8).

**Figure 9:**
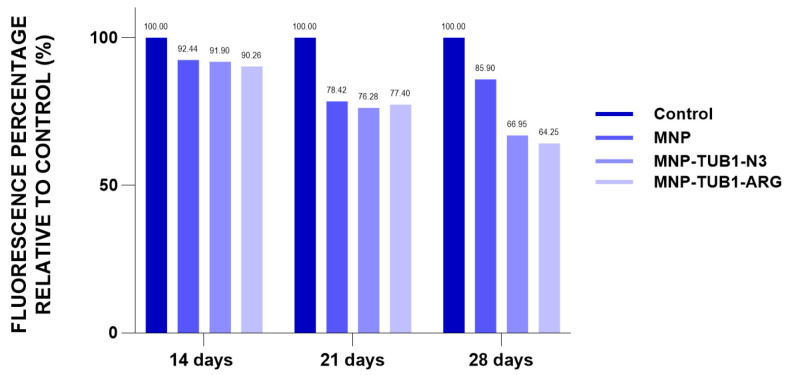
Time-dependent Beta-galactosidase activity per cell detected using fluorescent quantitative detection of enzymatic activity. Test performed for ADSC loaded with control formulations, magnetic nanoparticles (MNP and (NE:TUB1-N_3_-MNP and NE:TUB1-Arg-MNP), over one month (14, 21, 28 days).

In a separate experiment, we used fluorescent detection of β-Galactosidase (B-gal), a glycoside hydrolase enzyme whose activity increases in senescent cells and is currently used as a marker of cellular senescence. Compared to control cells that displayed time-dependent increased enzyme activity, ADSC incubated with as prepared MNP and especially with NE:TUB1-N_3_-MNP and NE:TUB1-Arg-MNP were found to limit enzyme activity in a time-dependent manner (Figure 9).

## 3. Discussion

Here, we report the successful formulation of senomodulator peptide nanoemulsions (NE:TUB1) that are stable, highly prone to large-scale manufacturing, and do not display cytotoxicity. For this study, a novel senolytic peptide, TUB1, modified with a tail of 8 arginine and azide group in its N-terminal, was successfully associated with sphingomyelin and vitamin E nanoemulsions by electrostatic interactions and covalent conjugation via copper-free “click” chemistry, respectively. Furthermore, we demonstrate that NE:TUB1-MNP formulations maintain stability and physical features (nanoscale dimension, homogeneity in emulsion, and peptide loading ability) and are non-toxic to cells in the short and medium term. Moreover, NE:TUB1-MNP was proven able to decrease enzymatic beta-galactosidase activity, counteracting culture-induced senescence detected in controls after 28 days of long-term culture.

The incorporation of the TUB1 peptide, modified with an arginine tail and an azide group, represents a novel approach to enhancing the peptide’s delivery and efficacy. The addition of the arginine tail likely improves the peptide’s cellular uptake, as polyarginine sequences are known to facilitate the translocation of peptides across cell membranes [38]. This modification could, on the one hand, allow the establishment of electrostatic interactions between NE and peptide and, on the other hand, enhance the intracellular delivery of TUB1, increasing its therapeutical potential. Moreover, the use of copper-free “click” chemistry for covalent conjugation of the peptide to the NE is a particularly innovative aspect of this study. Copper-free “click” chemistry is biocompatible, has high site specificity, and is a high-efficiency method. It not only ensures a strong, stable bond between the peptide and the NE but also preserves the bioactivity of TUB1, as the conjugation process does not involve harsh conditions that could denature the peptide. The association efficiency values were similar for both the “click” conjugation method (63.3 ± 5.9%) and electrostatic association (60.2 ± 10.9%), indicating that the covalent “click” chemistry approach does not offer a significant advantage over electrostatic interactions in terms of AE% for this particular NE:TUB1 system. Given the comparable performance and the faster process time, the electrostatic method may be more cost-effective. However, in terms of reproducibility, the “click” conjugation demonstrates higher consistency, with a standard deviation of 5.9 compared to 10.9 for electrostatic interactions. Nonetheless, both AE% values are consistent with previously reported studies employing similar strategies [24,38,39,40], indicating that a significant portion of the peptide is successfully associated with both methods. Therefore, the choice of conjugation strategy for this peptide should consider factors such as stability or release profile, as the AE% is similar across methods.

Positive-charged peptide nanoconjugates offer promising advantages for treating OA; these nanoconjugates, both exhibiting a positive charge (Table 1), may enhance targeting capabilities towards the ECM and improve uptake by chondrocytes through electrostatic attraction. Research indicates that nanoparticles with a positive charge, within an optimal range, demonstrate superior cellular uptake and diffusion within cartilage tissues, making them efficient carriers for therapeutic agents such as insulin-like growth factor 1 (IGF-1) [41]. Consequently, these nanoconjugates can be engineered to target specific tissues, like cartilage, improving penetration and retention within the articular space and enhancing the precision and efficacy of drug delivery in OA treatment.

One of the most critical aspects of nanoparticle formulation is ensuring stability, both in terms of physical properties and functional efficacy [42]. The formulations demonstrated stability in water under storage conditions (at 4 °C) for up to 25 days (Figure 2) and in a complete cell culture medium (at 37 °C) for up to 24 h (Figure 2) while maintaining their nanoscale dimensions and homogeneity, thereby ensuring consistent peptide delivery. Regarding surface charge, the difference between NE:TUB1-N_3_ (+38 mV) and NE:TUB1-Arg (+23 mV) can be attributed to their different conjugation methods. Covalent conjugation via “click” chemistry may orient the peptides in a way that exposes more charged residues to the surface, while electrostatic interactions might allow for more random orientations. Furthermore, covalent bonds are more stable than electrostatic interactions and, thus, could result in less dissociation of the peptides from the surface, maintaining a higher charge density. The sudden size increase observed for NE:TUB1-Arg after the first 30 min and for NE:TUB1-N_3_ during the first 6 h of incubation with non-supplemented cell culture medium (Figure 2) could be attributed to a destabilization of nanoemulsion upon contact with certain components of the medium, and/or a swelling process. The different behaviors observed between the two formulations could be linked to their distinct conjugation methods used (electrostatic vs. “click” chemistry), which may affect how the particles interact with components in the medium. Moreover, the fact that this size increase occurs rapidly suggests that it could be attributed to immediate interactions with the medium and discard possible long-term degradation processes. Conversely, both formulations remained stable in size after 24 h of incubation in a supplemented cell culture medium with fetal bovine serum. In this case, the formation of the protein corona may increase stability. This protein layer on the nanoparticle’s surface can act as a stabilizer by providing steric stabilization, reducing surface energy, and preventing particle–particle interaction and aggregation [42]. This is a positive finding, as the formulations maintain their integrity and size distribution under physiological conditions, which is crucial for potential biomedical applications.

This consistency is vital for therapeutic applications, where uniformity in size and peptide distribution directly influences the efficacy and safety of the treatment. Furthermore, the formulation’s stability over time indicates its suitability for long-term storage and use, an essential requirement for large-scale manufacturing and distribution. In this sense, the robust nature of the nanoemulsion formulation strategy suggests that they could be produced in large quantities, making them viable candidates for widespread therapeutic use. After obtaining stable NE:TUB1 formulations, we designed and characterized a formulation that includes proprietary MNP, aiming for increased efficiency and potentially inferring traceability to the final product. Consistent with previous reports, including MNP in the formulation does not induce cytotoxicity in the short, medium, or long term, as detected by evaluating two crucial mechanisms of cell survival: mitochondrial metabolism and membrane transport. Furthermore, MNP presence slightly increased cell viability compared to NE, NE:TUB1, and controls, likely due to increased iron availability that supports cell proliferation [43]. The non-cytotoxic nature of the NE is another major strength is this study. In vitro and in vivo applications of nanomaterials often raise concerns about potential toxicity, especially when dealing with complex formulations involving bioactive peptides and nanoparticle carriers [44,45,46]. The absence of cytotoxic effects in the short and medium term, as demonstrated in this study, is a promising indicator that the NE:TUB1 could be safely applied in therapeutic contexts without inducing adverse cellular responses.

As expected, membrane integrity in cells loaded with NE:TUB1-MNP was preserved, as confirmed by the LIVE/DEAD assay. Both MNPs and NE-MNPs are internalized through active membrane processes, such as endocytosis or pinocytosis, depending on particle size and tendency to aggregate. Given that the dynamic size of our formulation averages 120 nm, similar mechanisms of particle uptake are expected for NE, NE:TUB1, and NE:TUB1-MNP. Our previous studies have demonstrated that MNP uptake occurs via macropinocytosis in mesenchymal cells and mesenchymal stem cells, which possess macrophage-like functions comparable to those of synovial fibroblasts. No evidence of nuclear membrane disruption or translocation was observed using fluorescently labeled NE and NE:TUB1. The inclusion of MNP is intended to confer regenerative properties to the therapeutic NE:TUB1 formulation, particularly for intra-articular delivery.

Previous research has shown that MNPs can improve bone regeneration and fracture healing by promoting osteogenesis in mesenchymal stem cells and supporting mineralized matrix deposition. Mechanisms of MNP-induced bone repair include RUNX-2 activation, inhibition of osteoclastogenesis, and reduced apoptosis [47,48]. Furthermore, the activation of mechanosensitive TREK ion channels [49] suggests a potential role for MNP in repairing articular bone components damaged by OA. MNPs have also been shown to enhance the chondrogenic differentiation of mesenchymal stem cells (MSCs), with or without magnetic field exposure, by increasing the deposition of mature cartilage ECM components, such as collagen type II and aggrecan, while reducing matrix mineralization (collagen X) [50]. Intra-articular delivery of MSCs pretreated with iron and ascorbic acid has been found to improve articular cartilage repair in a minipig model of cartilage defects, as evidenced by MRI cartilage damage scores, highlighting the role of iron supplementation in joint surface recovery [51]. Importantly, the combination of MNP uptake and antioxidant payloads has been shown to modulate the inflammatory response in human adult stem cells, particularly in terms of cytokine release (Il-6, IL-1alpha), and to mitigate the response of activated peripheral blood monocytes to allergen preconditioning in vitro [52]. The use of MNPs and magnetic field exposure offers a potential approach to modulate cell signaling in vivo, raising the possibility of remotely controlling cell inflammatory responses and potentially influencing SASP [53]. Furthermore, iron oxide nanoparticles functionalized with the senolytic compound quercetin have been shown to protect human fibroblasts from culture-induced senescence via MAPK activation [54], indicating the viability of using MNPs as carriers for senomodulatory molecules. One of the key advantages of using NE:TUB1-MNPs formulations is their magnetic properties and responsiveness to external magnetic fields. We have confirmed the strong magnetic responsiveness of NE:TUB1-MNP’s, as evidenced by hysteresis loops that show remanence magnetization, indicative of ferromagnetic behavior (Figure 3). Additionally, we demonstrated that ADSCs can efficiently internalize NE:TUB1-MNP complexes and accumulate them intracellularly without the need for additional uptake enhancers. Simply adding the complexes to the culture media is sufficient for both coated and uncoated MNPs to be taken up by various mesenchymal cell types within the first 48 h of interaction, consistent with previous findings [55].

The intracellular iron levels detected by the ferrozine assay suggest that peptide addition facilitates this process, resulting in an average iron load of 6–7 picograms per cell. Given that synovial fibroblasts are likely to internalize the therapeutic formulation, this iron load could enable in vivo detection using MRI tracking systems, a possibility that warrants further investigation [56]. Previous studies have shown that MNP functionalization with cationic peptides enhances cellular uptake and prevents rapid intracellular degradation, leading to sustained payload delivery. The improved cellular uptake is likely due to the morphology, absence of toxicity, and the synergistic effect of MNP and cationic peptides [57]. Our previous data confirm that intracellular iron levels of 7–12 picograms per cell are sufficient to retain the stem cell phenotype, and these cells can be tracked both in vitro [58] and in vivo using clinically available MRI equipment. Therefore, we propose that intra-articular delivery of NE:TUB1-MNP formulations could enable medium- and long-term product traceability within the joint.

Beta-galactosidase activity is widely used as a marker for cellular senescence both in vitro and in vivo. The enzyme is selectively induced in senescent cells and is minimally expressed in proliferating or quiescent cells, making it a reliable indicator for detecting senescence. We previously demonstrated that MNP internalization by MSCs prevents culture-induced senescence. However, this effect was found to be cell type-dependent, as ADSCs, but not Wharton Jelly mesenchymal stem cells (WJMSC), exhibited reduced beta-galactosidase activity [58]. Here, we demonstrated that the presence of senolytic peptide decreased beta-galactosidase activity compared to controls and to ADSC loaded with MNPs alone. Furthermore, the superiority of the senolytic peptide became increasingly evident with extended culture time, confirming its utility in preventing culture-induced senescence in this in vitro model (Figure 9). Further tests and in vivo confirmation of these findings are needed. However, the current results provide solid evidence supporting the enhanced anti-senescent role of NE:TUB1-MNP, which warrants further investigation and potential exploitation.

The study presents compelling evidence that beta-galactosidase activity, a well-established marker of cellular senescence, is effectively modulated by specific interventions in MSCs. Our findings indicate that while MNP internalization by MSCs has been shown to prevent culture-induced senescence, this protective effect is not universally applicable across all MSC types. Specifically, ADSCs demonstrated a significant reduction in beta-galactosidase activity following MNP loading, whereas Wharton Jelly mesenchymal stem cells (WJMSC) did not exhibit a similar response [58]. This cell type-dependent variability underscores the importance of considering the biological context when developing anti-senescent strategies. In this context, the association with a senolytic peptide offered a novel approach to further mitigate senescence in MSCs. Our results reveal that the specific peptide not only decreased beta-galactosidase activity relative to untreated controls but also outperformed ADSCs treated solely with MNPs. The reduction in beta-galactosidase activity was even more pronounced with prolonged culture, highlighting the peptide’s sustained efficacy over time (Figure 9). This suggests that senolytic peptide can enhance the anti-senescent effects of MNPs, potentially offering a synergistic benefit in combating culture-induced senescence. The implications of these findings are significant, as they suggest a promising strategy for maintaining the viability and functionality of MSCs in culture, which is crucial for their therapeutic application. The superior performance of the senolytic peptide, particularly in combination with MNPs, supports its potential as a powerful tool in senescence prevention. However, it is important to acknowledge that while the in vitro results are promising, further investigation is needed to confirm these effects in vivo.

To summarize, this study provides a comprehensive in vitro characterization of magnetic nanoemulsions associating senolytic peptides that demonstrated biocompatibility and intracellular uptake properties, as well as the ability to target senescent cells effectively. Further studies are needed to explore the long-term effects of NE:TUB1-MNP in vivo, particularly in the context of chronic conditions where senescence plays a significant role. Although the TUB1 effect, in terms of protein expression levels, has already been studied and reported [17], understanding the precise mechanisms by which the peptide associated with the NE exerts its effect at the molecular level is still missing and would contribute to the development of even more effective senolytic therapies.

Present findings are encouraging, opening up the perspective of developing NE:TUB1-MNP-based solutions for treating OA in the future.

## 4. Materials and Methods

### 4.1. Materials

Egg Sphingomyelin (SM, Lipoid E SM, 98%) was purchased from Lipoid GmbH (Ludwigshafen, Germany). Vitamin E, (±)-α-tocopherol (VitE, 96%), 1,2-distearoyl-sn-glycero-3-phosphoethanolamine-N-[(Methoxylpolyethylene glycol)-2000] (DSPE-PEG-2k) was obtained from Nanocs (New York, NY, USA). Polyethylene glycol analog compound modified with DBCO (DSPE-PEG-2k-DBCO) was acquired from Avanti Polar Lipids (Alabaster, AL, USA). Absolute ethanol (EtOH, 99.7%) was acquired from VWR Chemicals (Radnor, PA, USA). MilliQ^®^ water (Millipore, 18.2 MΩ cm resistance) was used in this study. The senolytic peptide sequence was provided by the group led by Dr. Mayán (SEQ ID TUB1: N’-FKGVKDRVKGK-C’). All other chemicals and reagents were of analytical grade and used without further purification.

### 4.2. Methods

#### 4.2.1. Peptide Synthesis and Chemical Modification

These experiments used two peptide sequences composed of 11 amino acids: TUB1-Arg and TUB1-N_3_. Both were synthesized by Peptide Protein Research Ltd. (Funtley, Fareham, UK) and ordered with two modifications in the N-terminus: eight residues of Arginine in the case of TUB1-Arg and azide group linked to a lysine in the case of TUB1-N_3_. The final products (MW 2511 g/mol and 1431.7 g/mol for TUB1-Arg and TUB1-N_3_, respectively) were provided as a lyophilized powder with a purity > 95% after their purification and characterization by HPLC-UV/Vis and ESI-MS.

#### 4.2.2. Preparation of Blank (NE) and Peptide-Functionalized Nanoemulsions (NE:TUB1)

Oil in water (O/W) nanoemulsions were prepared by ethanol injection following two strategies previously reported by our group with minor modifications [23]. Briefly, VitE (5 mg, 11.6 μmol, 90.1% mol), SM (0.5 mg, 0.7 μmol, 5.3% mol), and DSPE-PEG-2k (0.05 mg, 0.02 μmol, 0.1% mol) were dissolved with a 1:0.1:0.01 mass ratio in 100 μL of absolute ethanol. In the case of the nanoemulsions without peptide (NE), the organic phase was injected into 0.9 mL of MilliQ water using an insulin syringe (0.5 mL, 0.33 × 12 mm ICO.C.1). For the peptide association (NE:TUB1), two different strategies were carried out depending on the peptide modification. For the TUB1-Arg, an electrostatic interaction strategy was performed. It is a non-covalent union based on surface adsorption through electrostatic interactions created between charged molecules. In this case, the TUB1-Arg peptide, chemically conjugated to an 8-residues chain of Arginine’s, which increases its positive net charge, was associated with the blank NEs (negatively charged), following the methodology previously described [36] with minor modifications. Before the formulation was carried out, the association of TUB1-Arg peptide (net charge +12 at pH 7) to DSPE-PEG2k (negative charge) took place in aqueous solution in a ratio of 5:1 (PEG:TUB1) and kept under magnetic stirring for 30 min to promote the formation of electrostatic interactions. Once the PEG–Peptide complex was formed, the organic phase was prepared by adding VitE and SM at a molar ratio of 1:0.1 (VitE:SM) and injected into ultrapure water to formulate the NE associated with the peptide. For the TUB1-N_3_, a conjugation based on a copper-free “click” chemistry was established between the azide group contained in the N-terminus of the peptide and the triple bond of the cyclooctyne (DBCO) moiety included in the DSPE-PEG-2k terminal. Analogous to the first strategy and as described elsewhere [34,37], the covalent conjugation of the peptide to the NE took place in 2 steps. Firstly, aqueous solutions of TUB1-N_3_ and DSPE-PEG-2k-DBCO were mixed in a ratio of 5:1 (PEG:TUB1) and kept at room temperature for 6 h, followed by incubation at 4 °C overnight. After this, nanosystems were spontaneously formed by the ethanol injection method, incorporating VitE and SM in a molar ratio of 1:0.1, as previously described. The resulting peptide concentration in both nanosystems was 2.5 μM.

#### 4.2.3. Synthesis of Magnetic Nanoparticles

The synthesis and characterization of bare and oleic acid-coated Fe_3_O_4_ magnetic nanoparticles, prepared by the traditional co-precipitation method, are described elsewhere [29]. Briefly, Fe_3_O_4_ nanoparticles were synthesized by adding FeCl_2_·4H_2_O (1.1 g) and FeCl_3_·6H_2_O (3 g) to deionized water (10 mL). The obtained solution was added to 400 mL of deionized water, stirred for 20 min, and filtered through a 0.22 µm filter; NaOH (15 g) was slowly poured into the reaction medium. In an instant, the solution turned black. After 2 min, the heating was turned off and stirring continued for 60 min. Sonication was performed on the suspension for 1 h in an ultrasound bath. We washed the magnetic nanoparticles several times with ultra-pure water until the pH reached 6. Once the suspension (approximately 75–80 mL) had been obtained, it was homogenized in an ultrasonic homogenizer at 100% amplitude for 40 min. For functionalization of magnetite with oleic acid, 200 µL of oleic acid was added over magnetite powder and ultrasonicated for 30 min. The obtained oleic acid-loaded magnetite nanoparticles were washed 3–5 times with deionized water until pH ±7.

#### 4.2.4. Formulation of NE-MNPs and NE:TUB1-MNPs Complexes

**Figure 10:**
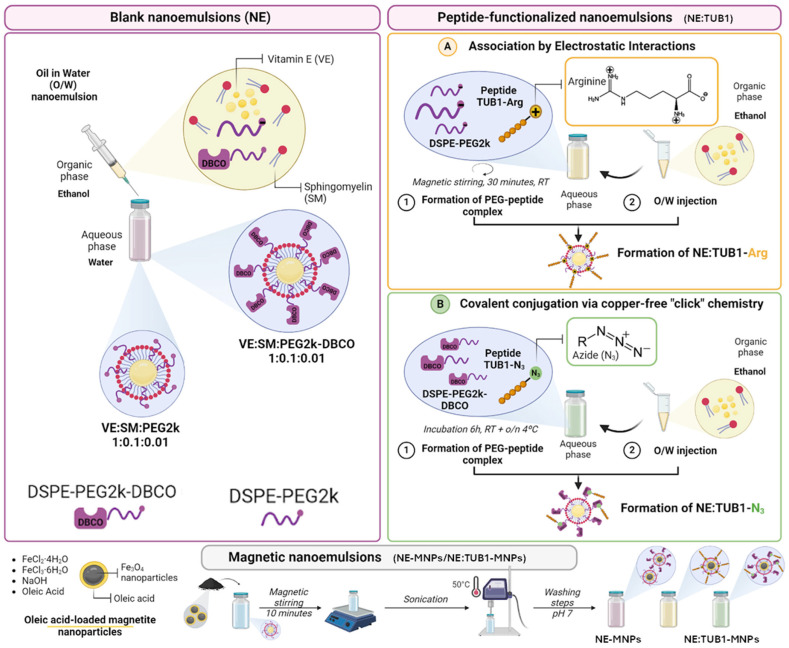
Scheme of blank and peptide-functionalized nanoemulsions preparation and Fe_3_O_4_ magnetic nanoparticle encapsulation. Abbreviations: Blank nanoemulsions composed of VitE:SM:PEG = NE; blank nanoemulsions encapsulating oleic acid-loaded magnetite nanoparticles = NE-MNP; peptide-functionalized nanoemulsions (without specifying peptide modification) = NE:TUB1; peptide-functionalized nanoemulsions (without specifying peptide modification) encapsulating oleic acid-loaded magnetite nanoparticles = NE:TUB1-MNP; peptide-functionalized nanoemulsions via covalent conjugation = NE:TUB1-N_3_; peptide-functionalized nanoemulsions via electrostatic interactions = NE:TUB1-Arg.

Magnetic nanoemulsions (NE-MNPs and NE:TUB1-MNPs) were prepared by mixing 500 µL of nanoemulsion (NE and NE:TUB1) and 100 µL of magnetic nanoparticles (MNPs, 40 µg/mL) under magnetic stirring for 10 min. The obtained magnetic-nanoformulation was sonicated in a UP50H ultrasonic processor (Hielscher Ultrasound Technology, Teltow, Germany) at a duty cycle of 70% and a frequency of 20 kHz, two times, for 4 min, with pauses of 2 min between sonication. A temperature of approximately 50 °C was reached during the sonication process. A graphical summary containing the formulation strategy is shown in Figure 10.

#### 4.2.5. Physicochemical Characterization

The nanoemulsions were physicochemical characterized by Dynamic Light Scattering (DLS) measurements using a NanoZS^®^, 676 nm laser (Malvern Instruments, Malvern, UK) at 25 °C and a scattering angle of 90°. The mean size and its distribution, defined by the polydispersity index (PDI) upon 10-fold dilution of the nanosystems in MilliQ water, reached a final lipid concentration of 0.5 mg/mL after 1:10 dilution of the samples in water.

Measurements were performed in disposable microcuvettes (ZEN0040, Brand, Los Angeles, CA, USA). The zeta potential (ZP) was analyzed by Laser Doppler Anemometry (LDA) diluting SNs 40-fold in MilliQ water (lipid concentration 0.12 mg/mL) in Folded capillary cuvettes (DTS 1070, Malvern Instruments). Short-term stability was performed for early determination of immediate instability behavior. Colloidal stability for up to 25 days was assessed after storing at 4 °C. Particle size and polydispersity index were determined according to the protocol previously detailed.

The particle size determination of magnetic nanoformulations was also measured using the dynamic light scattering method (DLS-Microtrac/Nanotrac 252, Microtrac Retsch GmbH, Montgomeryville, PA, USA) and ultra-high-resolution transmission electron microscopy imaging (Libra200 UHR-TEM, Carl Zeiss, Oberkochen, Germany). The magnetic properties of Fe_3_O_4_-NE (NE-MNPs) were determined by using a vibrating sample magnetometer (VSM) (LakeShore 7410). FTIR analysis (4000–400 cm^−1^) of the magnetic NEs was performed using the Fourier transform infrared spectroscopy, FTIR JASCO 6100.

The stability of NE and NE:TUB1 was tested upon incubation with culture medium DMEM at 37 °C for up to 24 h. The colloidal properties were measured by DLS, maintaining the same conditions mentioned before. Parameters such as the medium (water) and the temperature (25 °C) were fixed for all the measurements. Each sample performed at least 3 measurements, and the average of independent samples is provided (n = 3).

#### 4.2.6. Association Efficiency

The association efficiency (AE%) of TUB1 into the nanosystems was determined by direct quantification of the peptide using gradient HPLC analysis method following optimizations from other reported studies [33]. The isolation of nanosystems from free molecules was performed as follows: 100 µL of nanosystems (NE:TUB1-N_3_, NE:TUB1-Arg) were injected into dialysis cassettes (Slide-A-Lyzer™ MINI, Thermo Fisher Scientific, Waltham, MA, USA) with a membrane molecular-weight cut-off (MWCO) of 10 kDa. Then, the cassettes were immersed in 500 mL of ultra-pure water with gentle stirring at room temperature. The dialysis procedure was performed for 2 h first, then change water, and finally, another 2 h of dialysis and change water. The new volume of nanosystems was measured after dialysis. Before injecting the nanosystems in the HPLC column, they were dissolved in methanol (MeOH) at a volume ratio of 1:20 (NE:MeOH).

HPLC analysis was performed using an HPLC system 1260 Infinity II Agilent (Agilent Technologies, Santa Clara, CA, USA) equipped with a pump G7111A, an autosampler G7129A, and a UV–Vis detector G7114A set at 220 nm wavelength. An InfinityLab Poroshell 120 EC-C18 (100 mm × 4.6 mm, 4 µm pore size) Agilent column was used and operated at RT. The mobile phases used were (A) ultrapure water with 0.1% (*v*/*v*) TFA and (B) ACN with 0.1% (*v*/*v*) TFA. The following gradient conditions were used: 0 min B = 0%; 18 min, B = 80%; 20 min B = 0%, maintaining a flow rate of 1 mL/min. Standard calibration curves were linear in the range of 10–500 ppm.

The AE% of TUB1 peptide in the nanosystems was calculated using the following equation:

AE% = 100 × (Associated peptide µg)/(Total peptide µg),(1)

Equation (1): Direct measurement of the associated peptide fraction.

#### 4.2.7. Cells

##### Human Primary Adipose-Derived Mesenchymal Stem Cells (ADSCs)

Cells were extracted from human adipose tissue samples obtained from lipoaspirate derived from donors undergoing elective cosmetic procedures. Samples were collected after ethical board approval and patient informed consent. Samples were processed as previously described [29]. Briefly, the aspirate was washed three times with sterile phosphate-buffered saline (PBS) with 2% antibiotic and suspended in 0.1% collagenase (collagenase type I, Sigma Aldrich, Merck KGaA, Darmstadt, Germany). Enzymatic digestion was performed at 37 °C with 5% CO_2_ for 2–3 h with subsequent centrifugation at 300× *g* for 5 min followed by filtration (100 µm cell strainer). Pellets were suspended in complete culture media CCM (DMEM with 10% FBS and 2% antibiotic) and plated in size-appropriate culture flasks. ADSC in passages 3–5 were used for experiments.

##### Cell Viability

In vitro viability assay was conducted using the 5-dimethylthiazol-2-yl-2,5-diphenyltetrazoliumbromide (MTT) test (Vybrant MTT cell proliferation assay, ThermoFisher Scientific) following the manufacturer’s instructions. Dimethylsulphoxide (DMSO) was used as the diluting agent. The assay was performed after 24 h and after 7 and 21 days of cell interaction with NE, NE:TUB1, and NE:TUB1-MNP. Additionally, cytotoxicity tests were conducted using a concentration of 40 µg/mL MNPs after 48 h and after 14 and 21 days of interaction with ADSCs. Absorbance was measured at 570 nm using a Synergy HTX Multi-Mode Reader (BioTek). Cell viability (CV) was calculated using the formula:

CV = 100 × (Ods − ODb)/(Odc − ODb),(2)

where

ODs = Optical Density (OD) of particle-treated cells;
ODb = OD of the blank (media only);
ODc = OD of untreated cells.
Equation (2): Formula for calculating cell viability percentage.

##### LIVE/DEAD Assay

ADSCs were cultured in 24-well plates until 90% confluency. After 48 h, cells were treated with MNP, NE-MNP, and NE:TUB1-MNP suspensions and cultured further. After 7 days, cells were treated with a solution of 2 µM calcein. AM and 4 µM EthD-1 were added (0.5 mL in each well). Cells were incubated for 45 min at room temperature in the absence of light. Following incubation, cells were washed with PBS and observed with an EVOS inverted light microscope with red and green fluorescence filters (RFP for dead cells and GFP for live cells) and bright field (BF). The images presented are overlaid images of BF, RFP, and GFP.

##### Cellular Iron Uptake (Ferrozine Assay)

ADSC at passages 4–5 were seeded in 24 well plates at a density of 2 × 10^5^ cells per well. After 24 h, 20 µL of NE:TUB1-MNPs (constituting 10% of the total culture medium volume of 200 µL) were added to each well. After 48 h, the cells were washed twice with PBS to remove any extracellular iron. Subsequently, the ferrozine assay was performed to measure the iron content within the cells. Iron content in the cell lysates was quantified spectrophotometrically and normalized to the iron content of non-loaded control cells relative to cell number. A calibration curve was created using FeCl_3_ standards (0–300 µM) in 10 mM HCl, with absorbance measured at 550 nm on a microplate reader. The remaining wells were used for cell counting with a TC20 Automated Cell Counter (Bio-Rad, Hercules, CA, USA). The iron concentration was then normalized to the cell number per well.

##### Cell Morphology

Cell morphology and NE:TUB1 uptake were observed using an inverted fluorescence microscope EVOS using TopFluor labeled NE.

##### Beta-Galactosidase Activity (B-Gal)

Cells were seeded in 24 well plates at a density of 4 × 10^5^ cells per well. Beta-galactosidase enzyme activity was detected using the β-galactosidase (B-Gal) Detection Kit (Fluorometric) from Abcam (Cambridge, UK), following the manufacturer’s instructions. Briefly, after 14, 21, and 28 days of culture, the cells were lysed by four cycles of freezing and thawing. Fluorescein di-β-D-Galactopyranoside (FDG) was then added and incubated for 1 h at 37 °C. A stop buffer solution was added, and fluorescence was measured with a microplate reader (490–550 nm excitation/emission). Cell counts were performed at each time point from separate wells after trypsinization using a cell counter (as above).

## 5. Conclusions

We report the development of a novel nanoemulsion–senolytic peptide formulation enhanced with magnetic properties through the incorporation of magnetic nanoparticles. The resulting formulation, NE:TUB1-MNPs, can be produced using highly reproducible and straightforward procedures, facilitating scalable manufacturing. The formulations are homogenous, nano-sized, do not aggregate, display high stability when stored at normal temperatures, and are non-toxic to human primary cells. The cumulative senolytic effect of carried peptides has been proved in vitro through the assessment of enzymatic beta-galactosidase activity.

In conclusion, these formulations represent a significant advancement in anti-senescence therapy, particularly in the context of OA treatment. The stable, scalable, and non-cytotoxic nature of these senomodulator peptide nanoemulsions is notable. The innovative application of the TUB1 peptide, along with the development of various strategies for its association with the NE (both covalent and non-covalent conjugations) and the integration of MNPs, have shown promising anti-senescent effects. This multifaceted approach positions the formulation as a strong candidate for further development, potentially leading to significant progress in the treatment of OA and other joint-related conditions.

The novelty of this work lies in the innovative combination of peptide-functionalized nanoemulsions with magnetic nanoparticles for targeted intra-articular delivery, complemented by comprehensive characterization and safety evaluation.

## Figures and Tables

**Table 1:** Physicochemical characterization in terms of size, polydispersity index (PDI), and surface charge.

	Nanoemulsions (NE)	Size (nm)	PDI	Z-Potential (mV)	Peptide Concentration (µM)
Ø Peptide	VE:SM:PEG2k	72 ± 2	0.17 ± 0.02	−29.6 ± 4.3	-
With Peptide	NE:TUB1-Arg	151 ± 3	0.16 ± 0.02	+23.3 ± 5.0	99
Ø Peptide	VE:SM:PEG2k-DBCO	79 ± 10	0.20 ± 0.03	−20.6 ± 3.3	-
With Peptide	NE:TUB1-N_3_	143 ± 11	0.13 ± 0.06	+35.7 ± 3.8	167

## Data Availability

All data underlying the results are available as part of the article, and no additional source data are required.
